# Bilateral hypocalcemic cataract after total thyroidectomy in a young woman: case report

**DOI:** 10.1186/s12886-019-1224-9

**Published:** 2019-11-21

**Authors:** Kumale Tolesa Daba, Dagmawit Kifle Weldemichael, Gersam Abera Mulugeta

**Affiliations:** 1 Department of Ophthalmology, Jimma University, Jimma, Ethiopia; 2 Department of Surgery, Jimma University, Jimma, Ethiopia

**Keywords:** Hypocalcemia, Bilateral cataract, Total thyroidectomy, Hypoparathyroidism, Tetany, Serum calcium and Ethiopia

## Abstract

**Background:**

Hypocalcemia is a derangement in serum calcium level due to a vast spectrum of disorders, but the most common cause is surgery, usually thyroid gland surgery. Symptoms of hypocalcemia can be due to increased neuromuscular excitation resulting in tetany, paresthesia or seizure. It can also be because of deposition of calcium in soft tissues producing reduced vision /cataract or calcification of basal ganglia. Cataract is the most common ocular symptom of hypocalcemia.

**Case report:**

A twenty-six years old Ethiopian female patient presented with painless reduction of vision of both eyes. Five years prior to the reduction of vision she was diagnosed to have hypocalcemia. The serum calcium level was very low (3 mg/dl) due to damage to the parathyroid gland during total thyroidectomy for toxic goiter. She has been on supplemental calcium gluconate twice daily. She had typical bilateral symmetrical posterior sub capsular cataract with punctate iridescent opacities in the anterior and posterior cortex of the lens. Systemic examination revealed horizontal surgical scar on the anterior neck and positive Chvostek sign.

## Background

Thyrotoxicosis is a hyperthyroidism state where various symptoms occur due to a raised level of circulating thyroid hormones. There are four clinical types of the disease namely: diffuse toxic goiter (Graves’ disease), toxic nodular goiter, toxic nodule, hyperthyroidism due to rarer causes [1].

Diffuse toxic goiter and toxic nodular goiter with overactive inter-nodular tissue are treated with total thyroidectomy which cures by reducing the mass of overactive tissue [1]. Total thyroidectomy is associated with complications such as: hemorrhage, airway obstruction and recurrent laryngeal nerve injury with voice change. Other complications include: thyroid insufficiency, recurrence of thyrotoxicosis and secondary hypoparathyroidism which is transient (can present within 2–5 postoperative days) [1]. Hypoparathyroidism occurs due to removal of the parathyroid glands or infarction through damage to the parathyroid end artery [1]. Permanent hypoparathyroidism is not as such common occurring in less than 1% of cases [1]. It is associated with several systemic manifestations of hypocalcemia like tetany, confusion, muscle weakness and paresthesia. Hypocalcemic cataract is one of the long term consequences of hypocalcemia along with papilloedema, basal ganglia calcification, nephrocalcinosis and prolonged QT interval [1, 2]. We report a twenty-six years old Ethiopian female patient with bilateral cataract with a history of previous thyroid surgery and systemic hypocalcemic symptoms and signs.

## Case report

A 26 years old female patient came to Jimma University department of ophthalmology (JUDO) with a compliant of bilateral painless and progressive reduction of vision of 2 years duration. She underwent total thyroidectomy 7 years back for toxic diffuse goiter. A week after the surgery, she started to have circumoral numbness, paresthesia of the hands and legs, muscle cramp, stiffness of joints, mental confusion and irritability. Then she was diagnosed to have hypocalcemia due to damage to the parathyroid gland during the surgery and has been on supplemental calcium gluconate.

At the time of diagnosis her serum calcium level was very low (3 mg/dl) and she was started on calcium gluconate supplement 500 mg three times a day. Currently her serum calcium level raised to 8.4 mg/dl (8.2–10.4 mg/dl) after which she stopped using the supplement and was told to increase calcium reach foods only. She denied any history of trauma to the eye. Otherwise she has no other known systemic diseases like Diabetes Mellitus (DM), Hypertension (HTN) or Tuberculosis (TB).

**Fig. 1:**
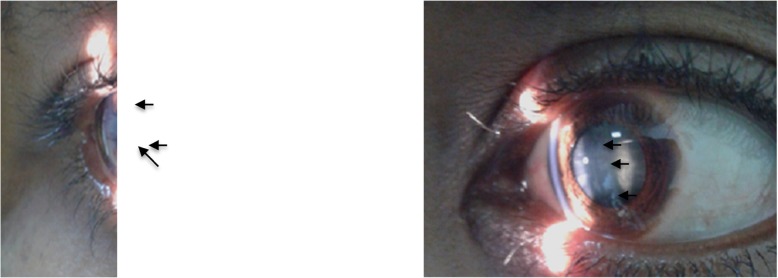
Iridescent opacities (black arrows) in the anterior and posterior cortex with posterior subcapsular cataract in both eyes

On Physical examination her visual acuity (VA) was 6/36 in both eyes. Intra Ocular Pressure (IOP) was 14 mmHg in the right and 12 mmHg in the left eye. Conjunctiva was quite, cornea was clear and transparent, anterior chamber (AC) depth was + 3 with Van Herrick (VH) classification and devoid of cells or flare. Pupil was round regular and reactive, there was no posterior psynechiae in both eyes. There was bilateraly symmetrical posterior sub capsular opacity and punctate iridescent opacities in the anterior and posterior cortex of the lens (Fig. 1). The fundus examination revealed no abnormality. On systemic examination she had horizontal surgical scar on anterior neck and she had Positive Chvostek sign.

The parathyroid hormone was significantly low (4.03 pg/mL, reference 10–65 pg/mL). The serum phosphate level was 6.5 mg/dl (reference 2.5–4.5 mg/dL). The level of serum magnesium was 1.8 mg/dl (reference 1.7–2.4 mg/dL). The level of troponin I was 0.01 ng/ml.

Other systemic Investigations revealed no remarkable finding. Complete Blood Count (CBC) was with in normal range. Erythrocyte Sedimentation Rate (ESR) was 30 mm/hr.; Fasting Blood Sugar (FBS) was 103 mg/dl. Venereal Diseases Research Laboratory (VDRL) and Provider Initiative HIV Counseling and Testing (PIHCT) were negative.

With the above findings she was diagnosed to have hypocalcemic cataract and we planned and counseled her to do phacoemulsification with Posterior Chamber Intra ocular Lens (PC-IOL). However the patient wanted to take some time to try alternative (religious prayer with Holy water) treatment. With the alternative treatment to date there was no change in the maturity of cataract.

## Discussion

Calcium regulation is critical for normal cell function, membrane stability, neural transmission, bone structure and blood coagulation. The normal total calcium concentration in the plasma is 8.9–10.1 mg/dL (4.5–5.1 mEq/L) [2]. Hypocalcemia is an electrolyte derangement commonly encountered on surgical and medical services [1, 2]. It may be transient, reversing with addressing the underlying cause, or chronic and even lifelong, when due to a genetic disorder or the result of irreversible damage to the parathyroid glands after surgery or secondary to autoimmune destruction [1–3]. After thyroidectomy, transient hypoparathyroidism occurs in 5–10% of patients; incidence of transient hypocalcaemia ranges 19–38% and permanent hypocalcemia occurs in 1% of patients [1].

Clinical symptoms of hypocalcemia can be due to increased neuromuscular excitation resulting in tetany, paresthesia or seizure [1]. It can also be because of deposition of calcium in soft tissues producing reduced vision due to cataract, papillaedema or calcification of basal ganglia [4–8]. It has been reported that longstanding hypocalcaemia has been remarkably asymptomatic or minimal symptoms like paresthesia [4]. Symptomatic long term hypocalcemia can occur after thyroid surgery with tetany, paresthesia, seizure and confusion [1, 2, 4]. The patient presented here had most of the above systemic manifestations which improved with supplemental calcium gluconate.

Hypocalcaemic cataracts are bilateral, punctate, iridescent opacities in the anterior and posterior cortex lying beneath the lens capsule which are usually separated from the lens capsule by a zone of clear lens. The opacities may remain stable or mature into complete cortical cataracts [3]. The morphology and bilaterality of the cataract with-out other concomitant primary ocular conditions in the case described here suggests the cause of the cataract to be long-term depreciation of serum calcium level in her body. The proposed mechanism of cataract formation in hypocalcemia is membrane damage with low calcium level in the aqueous humor and sodium content increase in the lens [9]. As far as the authors’ knowledge; there are few reported cases of bilateral cataract secondary to hypocalcemia [5, 6, 8]. Freedman and et al. reported bilateral cataract secondary to hypocalcaemia presenting 4 years after total thyroidectomy [5]. Our patient developed long-term hypocalcemia after total thyroidectomy which took longer time to return back to normal. Her serum calcium level at the time of diagnosis was very low. It took 7 years of treatment to raise her serum calcium level. Chronic hypocalcemia due to hypoparathyroidism is treated with calcium supplements (1000–1500 mg/d elemental calcium in divided doses) and either vitamin D2 or D3 (25,000–100,000 U daily) or calcitriol (1,25(OH)2D, 0.25–2 g/d) [2]. The patient described here has been on calcium gluconate 500 mg tid which is according to the recommended dose; was not taking vitamin D and calcitriol because they were not available in our setup and they are expensive to buy from abroad.

Other causes of bilaterally symmetrical cataract include metabolic disturbances like hyperphosphatemia, calcitonin reduction, vitamin D insufficiency and renal failure. Idiopathic hypoparathyroidism is another important cause of bilaterally symmetrical cataract. The patient presented here had a clear history of total thyroidectomy and was diagnosed to have hypocalcemia 7 years prior to her presentation to eye department. In addition the investigations were not suggestive of the above differential diagnoses.

## Conclusion

After thyroid surgery, permanent hypocalcemia should be considered as one of the long-term complications and patients should be followed for the symptoms of tetany and serum calcium levels. The work up of bilateral symmetrical cataract in young patients should include serum electrolyte levels, particularly calcium, in those who had history of thyroid surgery.

## Data Availability

Not applicable.

## Abbreviations

AC: Anterior Chamber
CBC: Complete Blood Count
DM: Diabetes Mellitus
ESR: Erythrocyte Sedimentation Rate
FBS: Fasting Blood Sugar
HIV: Human Immuno- deficiency Virus
HTN: Hypertension
IOP: Intra ocular pressure
JUDO: Jimma University Department of Ophthalmology
PC-IOL: Posterior Chamber Intra Ocular Lens
PIHCT: Provider Initiative HIV Counseling and Testing
TB: Tuberculosis
VA: Visual Acuity
VDRL: Venereal Disease Research Laboratory
VH: Van Herick
